# Benralizumab for Eosinophil-Related Cutaneous Adverse Events of Anticancer Therapy: A Phase II Trial

**DOI:** 10.1158/1078-0432.CCR-25-2764

**Published:** 2026-05-05

**Authors:** Mario E. Lacouture, Alexander Pan, Tara Maier, Anna Chen, George Dranitsaris, Neil J. Shah, Chau Dang, Devika Gajria, Allison Gordon, Neil Iyengar, Pedram Razavi, Mark Robson, Ezra Rosen, Serena Wong, Ucalene Harris, Kwami Ketosugbo, Cindy Bravo, Manu Jain, Alina Markova

**Affiliations:** 1 Dermatology Division, Department of Medicine, New York University Langone Hospital – Long Island, Garden City, New York.; 2 Dermatology Service, Department of Medicine, Memorial Sloan Kettering Cancer Center, New York, New York.; 3 Department of Public Health, Falk College, Syracuse University, New York, New York.; 4 Sidney Kimmel Center for Prostate and Urologic Cancers, Memorial Sloan Kettering Cancer Center, New York, New York.; 5 Evelyn H. Lauder Breast Center, Memorial Sloan Kettering Cancer Center, New York, New York.

## Abstract

**Purpose:**

Eosinophil-related cutaneous adverse events (ercAE) are common after systemic cancer therapies and often affect health-related quality of life (HRQoL). In this study, we investigate the efficacy and safety of benralizumab for ercAE following systemic cancer therapies.

**Patients and Methods:**

This single-arm, single-center, open-label, phase II trial (NCT04552288) in patients with cancer and systemic therapy-associated ercAE was performed from September 2020 to October 2023. Benralizumab 30 mg was administered subcutaneously according to the approved dosing schedule, with 3 doses given every 4 weeks followed by 3 doses given every 8 weeks. The primary endpoint was clinical response (reduction in ercAE to Common Terminology Criteria for Adverse Events grade ≤ 1 by week 4). Secondary endpoints included HRQoL, rash body surface area (rash-BSA), eosinophil levels, and AE.

**Results:**

At baseline (N = 47), ercAE were attributed to phosphoinositide 3-kinase inhibitors in 47%, checkpoint inhibitors in 21%, tyrosine kinase inhibitors in 9%, and antibody–drug conjugates in 9%; ercAE were grade 2 and 3 in 49% and 51% of patients, respectively. Of the 42 patients evaluable for the primary endpoint, 76% of patients (n = 32/42) responded to treatment by week 4; median ercAE grade decreased from 2 to 1 (*P* < 0.0001). Patients exhibited improved HRQoL, reduced mean rash-BSA, and decreased peripheral eosinophils. All patients with ercAE following alpelisib (n = 18) or enfortumab vedotin (n = 4) responded by week 4 (both *P* < 0.05). Most AE were mild to moderate and likely unrelated to benralizumab.

**Conclusions:**

Benralizumab demonstrated favorable efficacy and safety against grade 2/3 ercAE related to novel cancer therapies. Further investigation in larger placebo-controlled trials is warranted.

**Translational Relevance**

Eosinophil-related cutaneous adverse events (ercAE) from cancer therapies impact quality of life and dose intensity. Benralizumab is an afucosylated interleukin-5 receptor alpha–specific monoclonal antibody that depletes eosinophils. In this open-label, phase II study, we evaluated the efficacy and safety of benralizumab in managing ercAE. Overall, 76% of patients experienced a clinical response within 4 weeks of treatment with benralizumab. Most AE that emerged during the study were mild to moderate in severity and unlikely to be related to benralizumab. These findings suggest benralizumab’s potential as a safe and effective therapy for managing ercAE in patients with cancer, enabling them to continue life-prolonging cancer therapy while minimizing cutaneous toxicity. Larger studies with prophylactic and therapeutic benralizumab are warranted to further support these findings.

## Introduction

Eosinophils are associated with the development of adverse drug reactions (1, 2), with higher levels predictive of hospitalization and corticosteroid use (3). High levels of eosinophils and eosinophil-related cytokines have been observed in patients with immunotherapy-related AE (4–6). Furthermore, skin biopsy samples from patients with immunotherapy-related rashes have demonstrated eosinophilic infiltration (7, 8). These eosinophil-related cutaneous adverse events (ercAE) are common AE of anticancer therapies (9, 10). ErcAE may necessitate changes in treatment schedules. For example, phase I oncology trials of cytotoxic or molecularly targeted agents showed that ∼2% of patients develop grade ≥ 3 drug-related dermatologic AE, which would require anticancer therapy dose reduction (11). Furthermore, up to 5% of patients with severe ercAE must interrupt or discontinue anticancer treatment (12, 13), which may negatively affect outcomes. Although systemic corticosteroids may be effective in treating ercAE, their use can be limited by toxicities and a high rate of ercAE recurrence (10, 12, 14, 15). There are currently no Food and Drug Administration (FDA)–approved medications to treat ercAE.

ErcAE, type IVb reactions, are characterized by Th2-mediated immune responses and interleukin (IL) secretion (16); this includes IL5, a cytokine responsible for the proliferation, differentiation, and activation of eosinophils (16). Benralizumab is an afucosylated IL5 receptor alpha (IL5Rα)–specific monoclonal antibody, which is approved by the FDA for the treatment of severe eosinophilic asthma and eosinophilic granulomatosis with polyangiitis (EGPA; ref. 17). Benralizumab binds to IL5Rα and blocks IL5 interaction, thereby inhibiting IL5-mediated eosinophil differentiation/maturation (18). Additionally, the Fc domain of benralizumab binds to FcγRIIIa on NK cells, leading to eosinophil apoptosis via antibody-dependent cellular cytotoxicity (18). The result is depletion of eosinophils (19). Benralizumab has an oral corticosteroid-sparing effect in severe asthma and EGPA and a favorable safety profile in large-scale trials (20–24). This supports the investigation of benralizumab for the treatment of ercAE. Here, we evaluated the efficacy and safety of benralizumab in reducing symptoms and severity of ercAE related to checkpoint inhibitors (CPI), antibody-drug conjugates (ADC) and targeted therapies.

## Patients and Methods

### Study design and participants

This was a prospective, open-label, single-arm, phase II trial conducted at the Memorial Sloan Kettering (MSK) Cancer Center from September 2020 to October 2023 (NCT04552288). Potential participants were identified by a member of the patient’s treatment team, the principal investigator, or the research team. Participants were adults aged 18 to 85 years with solid or hematologic cancers (any stage). Eligible patients had developed grade 2/3 cutaneous toxicity [Common Terminology Criteria for Adverse Events (CTCAE) version 5.0; ref. 25] from systemic therapies, including immune CPI (i.e., ipilimumab, nivolumab, pembrolizumab, avelumab, durvalumab, atezolizumab, and tremelimumab) and/or targeted therapies [i.e., alpelisib, enfortumab vedotin (EV), everolimus, sorafenib, trastuzumab, pertuzumab, osimertinib, temsirolimus, and regorafenib], and had a blood absolute eosinophil count of ≥ 300 cells/μL. Patients with active helminthic infection and those who had received a live vaccine < 30 days before trial enrollment were excluded. Those receiving oral prednisone ≥ 20 mg daily at study start were also excluded as the dosing threshold was used in a previous benralizumab trial (21). However, the use of oral corticosteroids as additional supportive medication to manage ercAE was permitted after initiating study treatment. The study was approved by the MSK Institutional Review Board (IRB No. 20-344). The study was conducted in accordance with the ethical principles of the Declaration of Helsinki. All study participants provided written informed consent.

### Treatment

Eligible patients were dosed with benralizumab 30 mg subcutaneously by a healthcare professional once every 4 weeks for the first 3 doses (days 1, 28, and 56) and then once every 8 weeks for 3 subsequent doses (days 112, 168, and 224) or until lost to follow-up. The total treatment period was 36 weeks, including a 4-week follow-up after benralizumab treatment cessation. Patients continued to receive ercAE-causing anticancer treatment (culprit therapies) during the study.

### Trial endpoints

The primary endpoint was the percentage of patients with reductions in ercAE to grade ≤ 1 by week 4 of treatment. CTCAE grading was scored by one investigator for each patient visit. Secondary endpoints were changes in patient-reported outcomes on skin-related AE as measured by Skindex-16 (0 = “never bothered” to 100 = “always bothered”; ref. 26), rash body surface area (rash-BSA), blood and skin eosinophil levels, safety/tolerability of benralizumab, relative dose intensity (RDI) of anticancer therapy, and the need for additional medications to manage ercAE. The investigator attributed treatment-emergent AE to benralizumab as unrelated, unlikely related, possibly related, probably likely related, or definitely related based on the established FDA-approved adverse event profile of benralizumab and clinical judgment (17, 22, 23). Changes in cytokines and other inflammatory biomarkers that correlate with response to benralizumab were an exploratory endpoint. An investigator blinded to patient and study data measured the BSA of affected drug eruptions using 3D total-body photography images (27). A board-certified pathologist was blinded to patient and study data when counting eosinophils per high-power field (HPF) on hematoxylin and eosin–stained skin biopsy slides. Skindex-16 scores were further analyzed by subdomain scores: symptoms, emotions, and functioning.

### Statistical analysis

Patient demographic data, AE, clinicopathologic characteristics, and RDI were evaluated using descriptive statistics. A mixed model for repeated measures (MMRM) analysis was used to evaluate changes in ercAE grade, rash-BSA, Skindex-16 scores, levels of eosinophils in skin and blood, cytokine levels, and other inflammatory biomarkers over time in the entire study population. The Pearson correlation test was used to determine correlations between investigator-graded ercAE and blinded investigator-measured rash-BSA from 3D total body photography.

Post hoc analyses classified the patient population into five subgroups based on the culprit drugs: phosphoinositide 3-kinase (PI3K) inhibitors, CPI, antibody–drug conjugates (ADC), tyrosine kinase inhibitors (TKI), and other targeted therapies. Analyses were also conducted between responders to benralizumab and nonresponders. An MMRM analysis was used to evaluate differences in outcomes for the primary and secondary endpoints between subgroups. Logistic regression analysis was used to determine the association between the use of supportive medication and response to benralizumab in the total cohort or the association between the use of supportive medication and the culprit therapy subgroup. An original sample size of 28 patients was selected based on an MMRM analysis that estimated > 80% power to detect a significant difference, with an alpha level of 5%, if at least 17 out of 28 patients experienced a decrease in ercAE to grade ≤ 1. An expansion cohort of 19 patients was added after the initial 28 patients were accrued to increase the statistical power of the analysis. There were no adjustments for multiplicity. Statistical analyses were performed using Stata version 16.0 (StataCorp, LLC, RRID:SCR_012763).

## Results

### Patient population

**Figure 1.**
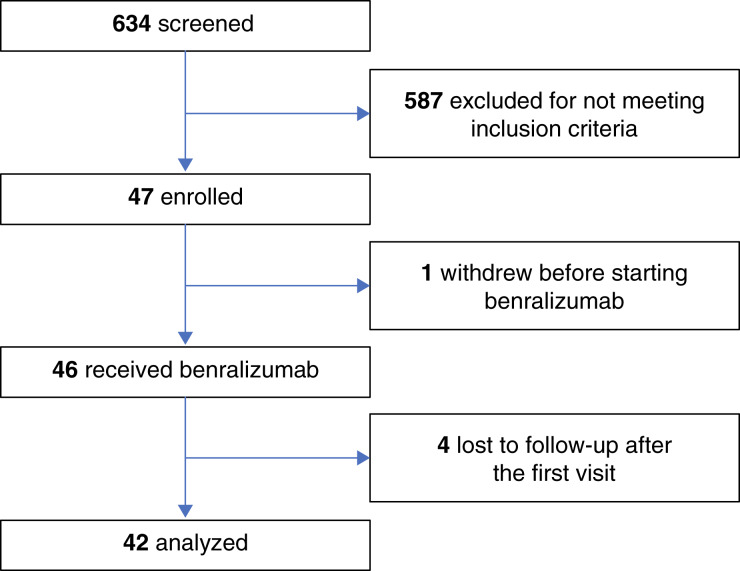
Study flow diagram.

Between September 2020 and October 2023, 47 patients were enrolled (Fig. 1). The mean age of enrolled patients was 61.2 years (SD, 11.7); 77% (*n* = 36) were White, 6% (*n* = 3) were Black, and 17% (*n* = 8) were Asian (Table 1; Supplementary Table S1). The predominant primary cancer diagnosis was breast cancer (*n* = 26, 55%), whereas others included bladder cancer (*n* = 5, 11%), melanoma (*n* = 5, 11%), kidney cancer (*n* = 3, 6%), and sarcoma (*n* = 3, 6%; Table 1).

**Table 1.** Baseline characteristics.

Baseline characteristics	All patients (*N* = 47)
Mean age (SD)	61.2 (11.7)
Sex, *n* (%)	​
Female	30 (64)
Male	17 (36)
Race, *n* (%)	​
White	36 (77)
Black	3 (6)
Asian	8 (17)
Ethnicity, *n* (%)	​
Hispanic or Latino	2 (4)
Non-Hispanic or Latino	45 (96)
Geography (%)	​
United States	100%
Primary malignancy, *n* (%)	​
Breast	26 (55)
Bladder	5 (11)
Melanoma	5 (11)
Kidney	3 (6)
Sarcoma	3 (6)
Lymphoma	2 (4)
Leukemia	1 (2)
Liver	1 (2)
Stomach	1 (2)
Dermatologic AE phenotype, *n* (%)^a^	​
Maculopapular rash	28 (60)
Pruritus	12 (26)
Eczema	5 (11)
Urticaria	3 (6)
Bullous pemphigoid	3 (6)
Lichenoid	1 (2)
Dermatologic AE grade (CTCAE), *n* (%)	​
2	23 (49)
3	24 (51)
Culprit therapy class, *n* (%)	​
PI3K inhibitor	22 (47)
CPI	10 (21)
TKI	4 (9)
ADC	4 (9)
MEK inhibitor	2 (4)
Anti-HER2 therapy	2 (4)
Anti-CD20 therapy	2 (4)
CDK4/6 inhibitor	1 (2)

Abbreviations: AE, adverse event; CD20, cluster of differentiation 20; CDK4/6, cyclin-dependent kinase 4 and 6; HER2, human epidermal growth factor receptor 2; MEK, mitogen-activated protein kinase; SD, standard deviation.

a Dermatologic AE phenotype category totals *n* = 52, which includes overlapping dermatologic phenotypes.

Culprit agents causing ercAE were immune CPI (*n* = 10, 21%) and targeted therapies (*n* = 37, 79%); the most common targeted therapies were PI3K inhibitors (*n* = 22, 47%, all alpelisib), ADC (*n* = 4, 9%, all EV), and TKIs (*n* = 4, 9%, imatinib and sorafenib; Table 1; Supplementary Table S2). At baseline, 23 (49%) patients had CTCAE grade 2, and 24 (51%) patients had CTCAE grade 3 skin conditions. Maculopapular rash was the most common phenotype (*n* = 28, 60%), followed by pruritus (*n* = 12, 26%), eczema (*n* = 5, 11%), urticaria (*n* = 3, 6%), bullous pemphigoid (*n* = 3, 6%), and lichenoid (*n* = 1, 2%; Table 1). No cases of drug reaction with eosinophilia and systemic symptoms were enrolled.

### Response to benralizumab

**Figure 2.**
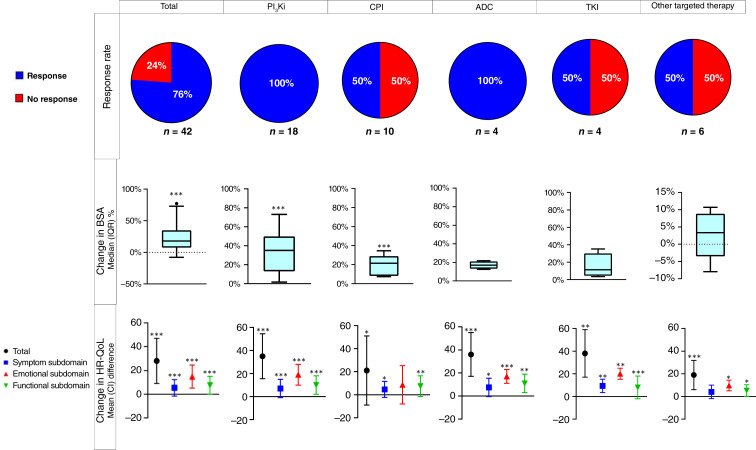
Clinical response to benralizumab^a^ in the total cohort and by culprit agent. Change in BSA and HRQoL from baseline to week 4. ^a^Response was defined as a reduction in ercAE to grade ≤ 1 by week 4. PI3Ki, PI3K inhibitor. *, *P* < 0.05; **, *P* < 0.01; ***, *P* < 0.001.

In total, 5 patients were not evaluable because they were either lost to follow-up after the first visit (*n* = 4) or withdrew before beginning protocol therapy (*n* = 1; Fig. 1). In the evaluable population of 42 patients, 32 (76%) had a response to benralizumab within 4 weeks after the first dose; in these patients, the median ercAE grade decreased from 2 to 1 by week 4 (*n* = 32, *P* < 0.0001). Of the 32 patients, 15 (47%) had a partial response (grade 1) and 17 (53%) had a complete response (grade 0) by week 4. All 22 patients with ercAE from alpelisib (PI3K inhibitor; *n* = 18) or EV (ADC; *n* = 4) had a response by week 4 (both *P* < 0.05; Fig. 2). In the alpelisib subgroup, the CTCAE grade decreased from a median of 2 at baseline to 0 at week 4 (*n* = 18, *P* < 0.001). Among the remaining patients, the response to benralizumab occurred in 5 out of 10 taking CPI, 2 out of 4 taking TKIs, and 3 out of 6 taking other targeted therapies (anti–human epidermal growth factor receptor 2, cyclin-dependent kinase 4/6 inhibitor, or mitogen-activated protein kinase inhibitor; Fig. 2).

Among the spectrum of clinical presentations of ercAE, maculopapular rash was the most common phenotype with a response to benralizumab (*n* = 19/25, 76%). Bullous pemphigoid, pruritus, eczema, urticaria, and lichenoid showed a response to benralizumab in 50% (*n* = 1/2), 64% (*n* = 7/11), 100% (*n* = 2/2), 100% (*n* = 1/1), and 100% (*n* = 1/1) of patients, respectively.

Overall, mean rash-BSA decreased between baseline and week 4 (from 37% to 12%, *P* < 0.0001). There were statistically significant correlations between investigator-graded CTCAE scores and blinded investigator-measured rash-BSA scores at week 4 (r^2^ = 0.40, *P* = 0.02) and week 8 (r^2^ = 0.55, *P* = 0.007).

### Health-related quality of life

The effect of skin AE on health-related quality of life (HRQoL) improved; from 0 to 2 weeks, following the first investigational agent administration, mean Skindex scores had reduced from 53.6 to 28.9 (*n* = 42, *P* < 0.001; Fig. 2). This decrease was maintained across subsequent time points, and responders in all culprit drug subgroups had significantly lower scores across questions assessing symptoms, emotions, and daily functioning (*n* = 42, *P* < 0.001 vs. week 0).

### Change in blood eosinophils and other biomarkers

**Figure 3.**
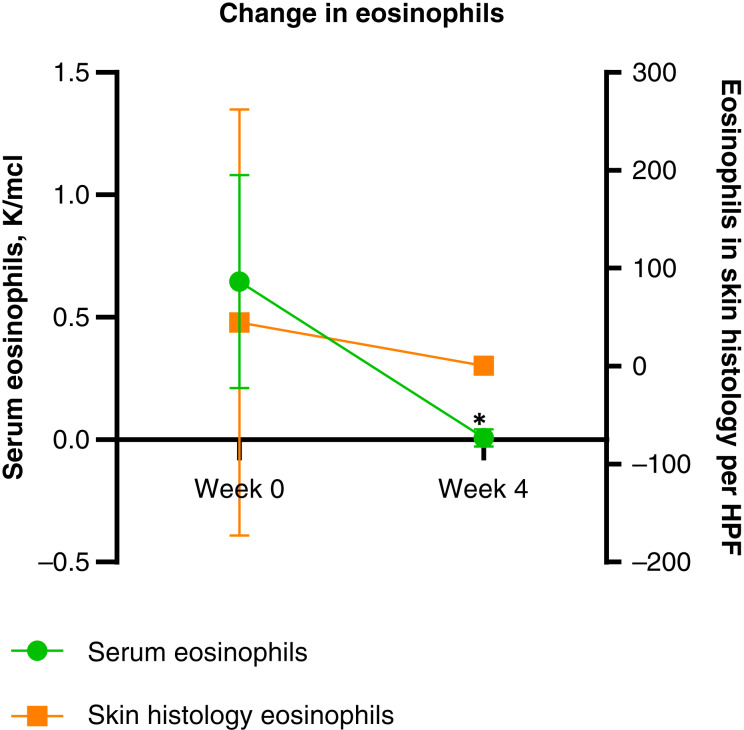
Change in serum and skin histology eosinophils in the total cohort. *, *P* < 0.05.

Independent of response, the level of peripheral eosinophils decreased in all patients, from a mean (SD) of 645 (436) cells/μL at baseline to 8 (50) cells/μL by week 4 (*n* = 42, *P* < 0.001; Fig. 3). There was no significant difference in eosinophil counts between different culprit drug subgroups.

**Figure 4.**
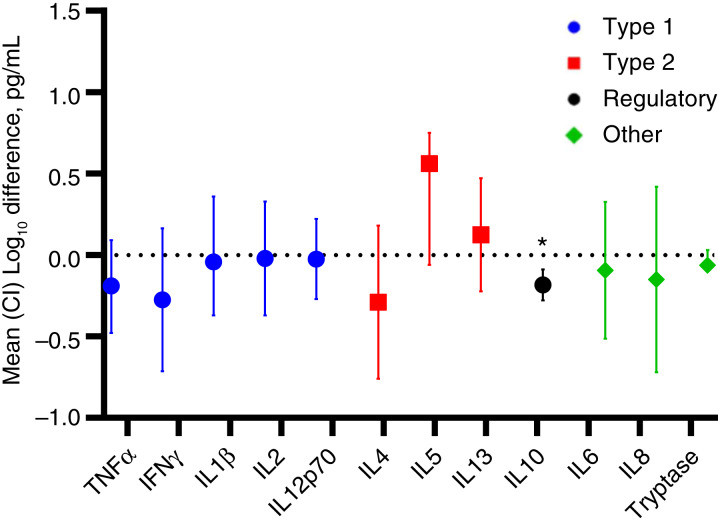
Change in biomarkers from baseline to week 4 in the total cohort. IFN, interferon; TNF, tumor necrosis factor. *, *P* < 0.05.

Serum cytokine levels were assessed to determine the effect of benralizumab on markers of inflammation (Fig. 4). TNFα, IL1β, IL2, IL4, IL5, IL6, IL8, IL12, IL13, tryptase, and IFNγ levels did not change significantly between baseline and week 4. Furthermore, cytokine levels were not significantly different between responders and nonresponders to benralizumab. IL10 levels significantly decreased between baseline and week 4 (mean, 2.3–0.7; *P* = 0.02), with 32 patients (76%) experiencing an absolute decrease in IL10 levels. Linear regression analysis revealed elevated tryptase levels in the ADC subgroup compared with the PI3K inhibitor subgroup [coefficient = 3.7; 95% confidence interval (CI), 1–6.3; *P* = 0.01].

### Histopathologic findings

Histopathologic samples of ercAE from baseline skin biopsies were analyzed from 43 patients. Histopathologic patterns included perivascular (*n* = 23, 53%), interface (*n* = 20, 47%), and spongiotic (*n* = 7, 16%) dermatitis. The most frequent epidermal changes included spongiosis (*n* = 14, 33%), keratinocyte necrosis (*n* = 6, 14%), parakeratosis (*n* = 6, 14%), and vacuolar changes (*n* = 5, 12%). Eosinophils and lymphohistiocytic infiltrates were present in 29 (67%) and 26 (60%) samples, respectively. Most histopathologic sample findings support the presence of a drug hypersensitivity reaction (*n* = 31, 72%). The other histopathologic reports supported broader diagnoses, which included urticarial reaction (*n* = 2, 5%), bullous pemphigoid (*n* = 2, 5%), lichen simplex chronicus (*n* = 1, 2%), eosinophilic folliculitis (*n* = 1, 2%), and nonspecific findings with clinical correlation advised (*n* = 5, 12%). There were no significant differences in the number of eosinophils in skin histology based on the culprit drug before treatment. After treatment, eosinophil numbers in skin samples were not significantly different between baseline and week 4 (mean, 92–0.37 per HPF; *P* = 0.2; Fig. 3; Supplementary Fig. S1). There was no significant association between the change in eosinophils in skin histology and clinical response.

### Concomitant supportive medications

Thirteen out of 42 patients (31%) used systemic corticosteroids during the study (Supplementary Table S3). All 13 patients who used systemic corticosteroids during the trial used prednisone ≥ 20 mg daily and did so before the week 4 primary endpoint. Seven patients (54%), 5 patients (38%), and 1 patient (8%) were prescribed prednisone ≥ 20 mg daily with taper for < 2 weeks, 2 to 4 weeks, and > 4 weeks, respectively. There was no significant difference in systemic corticosteroid use between responders and nonresponders to benralizumab in the total cohort, and no statistically significant difference in systemic corticosteroid use between culprit drug subgroups. In the alpelisib and ADC subgroups, systemic corticosteroids were used by 44% (*n* = 8/18) and 0% (*n* = 0/4) of patients, respectively (Supplementary Table S3).

Twenty-seven (64%) patients used topical corticosteroids during the study (Supplementary Table S3). Of the 27 patients who used topical steroids, specifically clobetasol propionate 0.05%, 25 (93%) were already using these therapies before the week 4 primary endpoint and were maintained on their treatment during the trial. Among these patients, 9 (33%) had been prescribed clobetasol for less than 2 weeks, 7 (26%) for 2 to 4 weeks, and 11 (41%) for more than 4 weeks. These patients had an inadequate response to topical steroid treatment alone, necessitating systemic therapy. Although patients were assigned a set duration for topical steroid use, they were instructed to apply the medication as needed, resulting in variability in usage among individuals. Drug diaries were not used in the study, limiting the ability to rigorously quantify the frequency and duration of adherence to topical steroid application. There was no significant difference in topical corticosteroid use between responders and nonresponders to benralizumab in the total cohort. Between culprit drug classes, patients receiving ADC used topical corticosteroids more frequently than those receiving alpelisib [odds ratio (OR), 4; 95% CI, 1.1–14.5; *P* = 0.04].

Fourteen patients (33%) receiving prophylactic antihistamines at baseline subsequently developed grade 2/3 ercAE from culprit therapy. In the total cohort, responders to benralizumab were less likely to use antihistamines during the study compared with nonresponders (OR, 0.15; 95% CI, 0.03–0.89; *P* = 0.04).

Between culprit drug classes, pregabalin for pruritus was used more frequently in patients receiving ADC (OR, 40.6; 95% CI, 2.8–598.1; *P* < 0.01) and TKI (OR, 20.8; 95% CI, 1.4–307.4; *P* = 0.03) than in those receiving alpelisib (Supplementary Table S3).

### Impact on cancer therapy

There was no significant difference in RDI between responders and nonresponders to benralizumab (median 92% and 95%, respectively, *P* = 0.23). Patients on PI3K inhibitors and CPI had the highest RDI (median 92% and 100%, respectively). RDI was higher in patients taking PI3K inhibitors and CPI than in those taking ADC (*P* < 0.05; Supplementary Fig. S2).

### Safety profile and patient-reported AE

Overall, 30 patients (71%) had AE during the study, 12 (29%) of which were AE grade ≥3 (Table 2). Mild-to-moderate mucositis, xerosis, and diarrhea were the most common AE. All AE were attributed as unrelated to investigational therapy. Serious AE were attributed to cancer therapy (*n* = 4, 33%), complications from primary malignancy (*n* = 4, 33%), infections in the setting of concomitant immunosuppressive agents (*n* = 2, 17%), exacerbation of comorbidity (*n* = 1, 8%), and myocardial infarction (*n* = 1, 8%). Deaths in the study were attributed to complications from primary malignancy (*n* = 2, 17%), infections in the setting of concomitant immunosuppressive agents (*n* = 1, 8%), and myocardial infarction (*n* = 1, 8%). There were no reported hypersensitivity reactions, injection-site reactions, pharyngitis, or headache with benralizumab.

**Table 2.** Adverse events.

Number (%) of patients	All patients (*N* = 42)	
Overview of AE	Any grade	Grade ≥ 3
Total	30 (71)	12 (29)
Serious AE	12 (29)	12 (29)
Death	4 (10)	4 (10)
Frequent AE reported in ≥ 10% or grade ≥ 3 reported in ≥ 3% of patients		
Mucositis	12 (29)	1 (2)
Xerosis	8 (19)	0 (0)
Diarrhea	8 (19)	0 (0)
Fatigue	4 (10)	4 (10)
Vomiting	3 (7)	2 (5)

Abbreviation: AE, adverse events.

## Discussion

Results of this study indicate that benralizumab mitigates grade 2/3 ercAE due to cancer therapies and has a favorable safety profile. Among evaluable patients, most (76%) responded to benralizumab by week 4. A reduction in rash-BSA positively correlated with response (r^2^ = 0.40, *P* = 0.02), and HRQoL improved in the whole group (mean Skindex-16 score, 53.6–28.9; *P* < 0.001). AE of interest reported in previous benralizumab clinical trials (headache, pyrexia, pharyngitis, and hypersensitivity reactions; ref. 17) were not reported by any patients. Overall, less than one-third of patients required additional systemic corticosteroids for the treatment of ercAE, and there was no significant difference in systemic corticosteroid use between responders and nonresponders in the total cohort. However, responders used antihistamines less frequently than nonresponders. There was a significant decrease in circulating IL10 during benralizumab treatment.

All 18 patients receiving alpelisib had a response to benralizumab; as this drug subgroup was the largest investigated in this study, these results suggest benralizumab may be especially effective in abating ercAE in patients treated with alpelisib and related agents. The group receiving alpelisib also had an improvement in HRQoL. Patients with eosinophilic rash due to inhibition of PI3K signaling had responses to benralizumab; this may shed light on the etiology of alpelisib-induced rash. Our results corroborate a retrospective study, in which increased peripheral eosinophils were found in patients with alpelisib rash (28). Furthermore, drug-induced maculopapular exanthems are typically characterized by blood eosinophilia and eosinophilic dermal infiltrates (29).

Among patients on alpelisib–fulvestrant in the phase III SOLAR-1 trial, AE related to hyperglycemia occurred in 66% of patients (grade ≥ 3 in 38%; ref. 30). AE related to rash were also common, occurring in 54% of patients on alpelisib–fulvestrant (grade ≥ 3 in 20%); 3% of patients discontinued alpelisib due to rash (30). A systematic review previously suggested that the incidence of PI3K inhibitor–associated cutaneous AE is approximately 29% (31). Oral corticosteroids are the standard treatment for grade 3 rash due to alpelisib (32); however, they can exacerbate alpelisib-induced hyperglycemia (33). In such cases, interruption or discontinuation of alpelisib is indicated, depriving patients of effective anticancer therapy (32). Benralizumab is a steroid-sparing agent (21, 24) that could allow patients to continue receiving anticancer therapy while minimizing hyperglycemia from steroids.

All four patients receiving EV responded to benralizumab by week 4. This is encouraging, considering the current management of EV rash is limited to systemic corticosteroids, antihistamines, and topical steroids (34), and EV-induced rash may cause changes in treatment schedules. For example, in the phase III EV-301 trial, treatment-related maculopapular rash occurred in 16% of patients receiving EV, with 4% requiring a dose reduction (35). Despite encouraging response rates in our study, the sample size was small (*n* = 4). The median RDI in the ADC group was 55%, the lowest among culprit therapy subgroups. Pregabalin was also used more frequently in the EV subgroup than in other subgroups. Consequently, further investigation is required to determine whether benralizumab can decrease dose reductions, interruptions, and discontinuations of EV therapy.

Among patients treated with anticancer-targeted therapies and CPI, benralizumab demonstrated moderate efficacy. It mitigated ercAE in 5 out of 10 patients receiving CPI, 2 out of 4 patients receiving TKIs, and 3 out of 6 patients receiving other targeted therapies and improved Skindex-16 in these subgroups by week 4, attesting to better HRQoL after treatment. Overall, 3 patients receiving CPI and 2 receiving TKIs or other targeted therapies required systemic corticosteroid support for rash management. Benralizumab may be another treatment option for immune-related cutaneous AE with associated eosinophilia, especially those refractory to systemic corticosteroids (10).

Despite reports suggesting a correlation between levels of peripheral eosinophilia and cutaneous toxicity induced by anticancer therapies (2), in the current study, depletion of eosinophils did not lead to responses in all cases. One possible explanation for this is the persistence of eosinophil granule proteins in skin tissue after eosinophil depletion. In a retrospective study of patients with eosinophilic esophagitis (EoE), esophageal biopsies were examined using immunostaining for eosinophilic major basic protein 1 (eMBP1, an eosinophil granule protein) and peak eosinophil counts in histopathology (36). There was a correlation between EoE symptoms and higher grades of eMBP1, but not peak eosinophil counts (36). Remnant eMBP1 and other eosinophil granule proteins potentially contributed to persistent cutaneous toxicity in benralizumab nonresponders, although further analysis of skin infiltration of granule proteins is needed before firm conclusions can be proposed.

The duration of ercAE varies widely across different classes of culprit agents based on previously published data. Alpelisib-associated rash typically has a short duration of approximately 1 week when managed appropriately. In a retrospective analysis of 102 patients, alpelisib rash lasted a mean duration of 7 days (28). In the clinical trial setting, 141 of 153 patients (92%) had complete resolution (32). However, the short duration of alpelisib-associated rash is primarily due to prompt interruption of therapy combined with aggressive medical management rather than spontaneous management during continued treatment. Specifically, for grade 3 rash, therapy interruption is standard, with corticosteroids and antihistamines used for management. According to FDA prescribing information, therapy is resumed at a lower dose once the rash improves to grade ≤ 1 (17). In a retrospective analysis, 84.2% of patients with grade 3 alpelisib-associated rash required alpelisib interruption, and 75% of those rechallenged after grade 3 rash did not experience recurrence, indicating that this interruption-based strategy is effective in controlling the rash (28). The use of prophylactic antihistamines reduces the incidence and severity of rash but does not eliminate the need for interruption in severe cases. Prophylactic antihistamines were associated with lower rates of all-grade rash (26.7% vs. 64.1%) and grade 3 rash (11.6% vs. 22.7%; ref. 32).

Cutaneous toxicities from enfortumab–vedotin generally resolve within 2 to 3 weeks when dose reductions are applied (35). Cutaneous toxicities from TKI last up to 3 weeks with concomitant treatment of systemic steroids (15). In contrast, cutaneous toxicities from CPI typically persist longer, with a reported median duration of 50 days (range, 1–352 days; ref. 37). Although the results observed in this study should be considered within the context of these varying timelines outside of the benralizumab treatment setting, it should be noted that the published data on cutaneous toxicity duration across different anticancer agents are influenced by management strategies and are not reflective of ercAE duration without any management.

### Limitations

Our study has several limitations; first, the single-arm, open-label design precludes attribution of outcomes to benralizumab due to the absence of a control group. Second, a relatively small sample size (*N* = 42 evaluable) limits power, particularly for subgroup analyses. Third, the heterogeneity of tumor types and culprit therapies may hinder the generalizability of the findings. Finally, the lack of blinding and randomization to standard treatments, such as systemic corticosteroids, limits the ability to ascertain the relative efficacy of benralizumab.

### Conclusion

Benralizumab demonstrated efficacy in treating PI3K inhibitor– and EV-induced ercAE, along with improvements in HRQoL and reductions in peripheral eosinophils. Further investigations in larger, placebo-controlled trials are required to evaluate the safety and efficacy of benralizumab for the treatment of ercAE induced by anticancer therapies.

## Supplementary Material

Supplementary Data1 Supplementary materials

## Data Availability

The data generated in the study are not publicly available due to patient privacy but are available from the corresponding author upon reasonable request.

## References

[1] Tracey EH , Modi B, Micheletti RG. Pemetrexed-induced pseudocellulitis reaction with eosinophilic infiltrate on skin biopsy. Am J Dermatopathol 2017;39:e1–2. 27415636 10.1097/DAD.0000000000000645

[2] Zhou J , Du Z, Fu J, Yi X. Blood cell counts can predict adverse events of immune checkpoint inhibitors: a systematic review and meta-analysis. Front Immunol 2023;14:1117447. 36960068 10.3389/fimmu.2023.1117447 PMC10029759

[3] Yang J , Yang X, Li M. Peripheral blood eosinophil counts predict the prognosis of drug eruptions. J Investig Allergol Clin Immunol 2013;23:248–55. 23964554

[4] Fujisawa Y , Yoshino K, Otsuka A, Funakoshi T, Fujimura T, Yamamoto Y, . Fluctuations in routine blood count might signal severe immune-related adverse events in melanoma patients treated with nivolumab. J Dermatol Sci 2017;88:225–31. 28736218 10.1016/j.jdermsci.2017.07.007

[5] Delyon J , Mateus C, Lefeuvre D, Lanoy E, Zitvogel L, Chaput N, . Experience in daily practice with ipilimumab for the treatment of patients with metastatic melanoma: an early increase in lymphocyte and eosinophil counts is associated with improved survival. Ann Oncol 2013;24:1697–703. 23439861 10.1093/annonc/mdt027

[6] Grisaru-Tal S , Rothenberg ME, Munitz A. Eosinophil-lymphocyte interactions in the tumor microenvironment and cancer immunotherapy. Nat Immunol 2022;23:1309–16. 36002647 10.1038/s41590-022-01291-2 PMC9554620

[7] Tetzlaff MT , Nagarajan P, Chon S, Huen A, Diab A, Omar P, . Lichenoid dermatologic toxicity from immune checkpoint blockade therapy: a detailed examination of the clinicopathologic features. Am J Dermatopathol 2017;39:121–9. 28134729 10.1097/DAD.0000000000000688

[8] Naidoo J , Schindler K, Querfeld C, Busam K, Cunningham J, Page DB, . Autoimmune bullous skin disorders with immune checkpoint inhibitors targeting PD-1 and PD-L1. Cancer Immunol Res 2016;4:383–9. 26928461 10.1158/2326-6066.CIR-15-0123 PMC5241697

[9] Chen CH , Yu HS, Yu S. Cutaneous adverse events associated with immune checkpoint inhibitors: a review article. Curr Oncol 2022;29:2871–86. 35448208 10.3390/curroncol29040234 PMC9032875

[10] Phillips GS , Wu J, Hellmann MD, Postow MA, Rizvi NA, Freites-Martinez A, . Treatment outcomes of immune-related cutaneous adverse events. J Clin Oncol 2019;37:2746–58. 31216228 10.1200/JCO.18.02141 PMC7001790

[11] Drilon A , Eaton AA, Schindler K, Gounder MM, Spriggs DR, Harris P, . Beyond the dose-limiting toxicity period: dermatologic adverse events of patients on phase 1 trials of the Cancer Therapeutics Evaluation Program. Cancer 2016;122:1228–37. 26916138 10.1002/cncr.29918 PMC5479632

[12] Geisler AN , Phillips GS, Barrios DM, Wu J, Leung DYM, Moy AP, . Immune checkpoint inhibitor-related dermatologic adverse events. J Am Acad Dermatol 2020;83:1255–68. 32454097 10.1016/j.jaad.2020.03.132 PMC7572894

[13] Naidoo J , Page DB, Li BT, Connell LC, Schindler K, Lacouture ME, . Toxicities of the anti-PD-1 and anti-PD-L1 immune checkpoint antibodies. Ann Oncol 2015;26:2375–91. 26371282 10.1093/annonc/mdv383 PMC6267867

[14] Kulkarni S , Durham H, Glover L, Ather O, Phillips V, Nemes S, . Metabolic adverse events associated with systemic corticosteroid therapy-a systematic review and meta-analysis. BMJ Open 2022;12:e061476. 10.1136/bmjopen-2022-061476 PMC9772659 36549729

[15] Kim EJ , Ryu MH, Park SR, Beck MY, Lee WJ, Lee MW, . Systemic steroid treatment for imatinib-associated severe skin rash in patients with gastrointestinal stromal tumor: a phase II study. Oncologist 2020;25:e1785–93. 32589310 10.1634/theoncologist.2019-0953 PMC7648351

[16] Ramirez GA , Yacoub MR, Ripa M, Mannina D, Cariddi A, Saporiti N, . Eosinophils from physiology to disease: a comprehensive review. Biomed Res Int 2018;2018:9095275. 29619379 10.1155/2018/9095275 PMC5829361

[17] U.S. Food and Drug Administration . Fasenra (benralizumab) prescribing information. 2024 [cited 2025 Dec 18]. Available from: https://www.accessdata.fda.gov/drugsatfda_docs/label/2024/761070s021lbl.pdf.

[18] Kolbeck R , Kozhich A, Koike M, Peng L, Andersson CK, Damschroder MM, . MEDI-563, a humanized anti-IL-5 receptor alpha mAb with enhanced antibody-dependent cell-mediated cytotoxicity function. J Allergy Clin Immunol 2010;125:1344–53.e2. 20513525 10.1016/j.jaci.2010.04.004

[19] Pelaia C , Calabrese C, Vatrella A, Busceti MT, Garofalo E, Lombardo N, . Benralizumab: from the basic mechanism of action to the potential use in the biological therapy of severe eosinophilic asthma. Biomed Res Int 2018;2018:4839230. 29862274 10.1155/2018/4839230 PMC5971345

[20] Criner GJ , Celli BR, Brightling CE, Agusti A, Papi A, Singh D, . Benralizumab for the prevention of COPD exacerbations. N Engl J Med 2019;381:1023–34. 31112385 10.1056/NEJMoa1905248

[21] Nair P , Wenzel S, Rabe KF, Bourdin A, Lugogo NL, Kuna P, . Oral glucocorticoid-sparing effect of benralizumab in severe asthma. N Engl J Med 2017;376:2448–58. 28530840 10.1056/NEJMoa1703501

[22] Bleecker ER , FitzGerald JM, Chanez P, Papi A, Weinstein SF, Barker P, . Efficacy and safety of benralizumab for patients with severe asthma uncontrolled with high-dosage inhaled corticosteroids and long-acting β_2_-agonists (SIROCCO): a randomised, multicentre, placebo-controlled phase 3 trial. Lancet 2016;388:2115–27. 27609408 10.1016/S0140-6736(16)31324-1

[23] FitzGerald JM , Bleecker ER, Nair P, Korn S, Ohta K, Lommatzsch M, . Benralizumab, an anti-interleukin-5 receptor α monoclonal antibody, as add-on treatment for patients with severe, uncontrolled, eosinophilic asthma (CALIMA): a randomised, double-blind, placebo-controlled phase 3 trial. Lancet 2016;388:2128–41. 27609406 10.1016/S0140-6736(16)31322-8

[24] Wechsler ME , Nair P, Terrier B, Walz B, Bourdin A, Jayne DRW, . Benralizumab versus mepolizumab for eosinophilic granulomatosis with polyangiitis. N Engl J Med 2024;390:911–21. 38393328 10.1056/NEJMoa2311155

[25] National Cancer Institute . Common Terminology Criteria for Adverse Events (CTCAE) Version 5.0. National Cancer Institute; 2017. [cited 2023 Nov 28]. Available from: https://ctep.cancer.gov/protocoldevelopment/electronic_applications/ctc.htm#ctc_50.

[26] Chren MM . The Skindex instruments to measure the effects of skin disease on quality of life. Dermatol Clin 2012;30:231–6, xiii. 22284137 10.1016/j.det.2011.11.003 PMC3269028

[27] Oh Y , Lacouture ME, Paras M, Kurtansky N, Kern J, Leung DYM, . Quantifying the clinical severity of immune-related cutaneous adverse events in clinical trial patients: a prospective study using 3D-total body photography. J Clin Oncol 2021;39:e13548.

[28] Wang DG , Barrios DM, Blinder VS, Bromberg JF, Drullinsky PR, Funt SA, . Dermatologic adverse events related to the PI3Kα inhibitor alpelisib (BYL719) in patients with breast cancer. Breast Cancer Res Treat 2020;183:227–37. 32613539 10.1007/s10549-020-05726-y PMC7398571

[29] Khandpur S , Ahuja R. Drug-induced vs. viral maculopapular exanthem-resolving the dilemma. Dermatopathology (Basel) 2022;9:164–71. 35645232 10.3390/dermatopathology9020021 PMC9149972

[30] André F , Ciruelos E, Rubovszky G, Campone M, Loibl S, Rugo HS, . Alpelisib for PIK3CA-mutated, hormone receptor-positive advanced breast cancer. N Engl J Med 2019;380:1929–40. 31091374 10.1056/NEJMoa1813904

[31] Jfri A , Meltzer R, Mostaghimi A, LeBoeuf N, Guggina L. Incidence of cutaneous adverse events with phosphoinositide 3-kinase inhibitors as adjuvant therapy in patients with cancer: a systematic review and meta-analysis. JAMA Oncol 2022;8:1635–43. 36227613 10.1001/jamaoncol.2022.4327 PMC9562095

[32] Rugo HS , André F, Yamashita T, Cerda H, Toledano I, Stemmer SM, . Time course and management of key adverse events during the randomized phase III SOLAR-1 study of PI3K inhibitor alpelisib plus fulvestrant in patients with HR-positive advanced breast cancer. Ann Oncol 2020;31:1001–10. 32416251 10.1016/j.annonc.2020.05.001

[33] Gallagher EJ , Moore H, Lacouture ME, Dent SF, Farooki A, Goncalves MD, . Managing hyperglycemia and rash associated with alpelisib: expert consensus recommendations using the Delphi technique. NPJ Breast Cancer 2024;10:12. 38297009 10.1038/s41523-024-00613-x PMC10831089

[34] Lacouture ME , Patel AB, Rosenberg JE, O’Donnell PH. Management of dermatologic events associated with the nectin-4-directed antibody-drug conjugate enfortumab vedotin. Oncologist 2022;27:e223–32. 35274723 10.1093/oncolo/oyac001 PMC8914492

[35] Powles T , Rosenberg JE, Sonpavde GP, Loriot Y, Durán I, Lee JL, . Enfortumab vedotin in previously treated advanced urothelial carcinoma. N Engl J Med 2021;384:1125–35. 33577729 10.1056/NEJMoa2035807 PMC8450892

[36] Peterson KA , Gleich GJ, Limaye NS, Crispin H, Robson J, Fang J, . Eosinophil granule major basic protein 1 deposition in eosinophilic esophagitis correlates with symptoms independent of eosinophil counts. Dis Esophagus 2019;32:doz055. 31310661 10.1093/dote/doz055

[37] Patel AB , Farooq S, Welborn M, Amaria RN, Chon SY, Diab A, . Cutaneous adverse events in 155 patients with metastatic melanoma consecutively treated with anti-CTLA4 and anti-PD1 combination immunotherapy: incidence, management, and clinical benefit. Cancer 2022;128:975–83. 34724197 10.1002/cncr.34004

[38] DeTora LM , Toroser D, Sykes A, Vanderlinden C, Plunkett FJ, Lane T, . Good Publication Practice (GPP) guidelines for company-sponsored biomedical research: 2022 update. Ann Intern Med 2022;175:1298–1304. 36037471 10.7326/M22-1460

