# Supplementary material for: Benralizumab for Eosinophil-Related Cutaneous Adverse Events of Anticancer Therapy: A Phase II Trial
Source: Clin Cancer Res. 2026 May 5;32(15):3195–202. doi: 10.1158/1078-0432.CCR-25-2764 (PMC13430216; doi:10.1158/1078-0432.CCR-25-2764)
Supplement: Supplementary Data1 — Supplementary materials [file ccr-25-2764_supplementary_data1_suppds1.docx]

**Supplementary Table 1:** Representativeness of Study Participants

| Cancer type(s)/subtype(s)/stage(s)/condition: | Eosinophil-related cutaneous adverse events caused by checkpoint inhibitors and anti-cancer targeted therapies in cancer patients. |
| --- | --- |
| Considerations related to: |  |
| Sex | Cutaneous adverse events from cancer therapies occur across both sexes, reflecting the underlying cancer populations treated with immune checkpoint inhibitors and targeted therapies. |
| Age | Mean age of developing immune-related cutaneous adverse events is 65 (range 18-89), reflecting the age of diagnosis of most solid tumor malignancies. |
| Race/ethnicity | Checkpoint inhibitor use is heavily concentrated in North America, Western Europe, and Japan. Access in underrepresented populations remains limited. |
| Geography | Checkpoint inhibitor use is heavily concentrated in high-income countries, notably in North America, Western Europe, and Japan. Access in low-income countries remains limited. |
| Other considerations: | Pivotal checkpoint inhibitor clinical trials have generally underrepresented non-White participants. There are limited data on sub-group analysis for cutaneous adverse events by race/ethnicity in these clinical trials. |
| Overall representativeness of the study: | The study’s population reflects the typical demographic profile of patients receiving immune checkpoint inhibitors and targeted therapies, with a mean age of 61 years, consistent with the age distribution of patients with solid malignancies reported in the literature.  However, this was a single-center study conducted at a tertiary care cancer center, primarily at the main site in New York City and affiliated satellite sites in Basking Ridge, New Jersey and Long Island, New York. As such, the study population was limited in geographic and ethnic diversity, which may affect the generalizability of the findings. |

**Supplementary Table 2.** Concomitant Cancer Medications

| **Cancer therapy regimen** | **Frequency (n, %)** |
| --- | --- |
| Alpelisib + Fulvestrant | 22, 47% |
| Enfortumab Vedotin | 4, 9% |
| Ipilimumab + Nivolumab | 3, 7% |
| Nivolumab | 2, 5% |
| Pembrolizumab | 2, 5% |
| Pertuzumab + Trastuzumab | 2, 5% |
| Imatinib | 2, 5% |
| Binimetinib + Encorafenib | 2, 5% |
| Rituximab | 2, 5% |
| Cabozantinib + Nivolumab | 1, 2% |
| Nivolumab + Fluorouracil, leucovorin, oxaliplatin (FOLFOX) | 1, 2% |
| Durvalumab + Tremelimumab | 1, 2% |
| Bosutinib | 1, 2% |
| Sorafenib | 1, 2% |
| Abemaciclib + Fulvestrant | 1, 2% |

**Supplementary Table 3.** Concomitant Supportive Medication Use at Any Point During the Study

| **Concomitant supportive medication, n (%)** | **Culprit therapy class** | | | | | |
| --- | --- | --- | --- | --- | --- | --- |
|  | **All culprit therapy classes combined**  **(n = 42)** | **PI3K inhibitor**  **(n = 18)** | **CPI**  **(n = 10)** | **ADC**  **(n = 4)** | **TKI**  **(n = 4)** | **Other targeted therapy**  **(n = 6)** |
| Systemic corticosteroids | 13 (31) | 8 (44) | 3 (30) | 0 (0) | 1 (25) | 1 (17) |
| Antihistamines | 19 (45) | 11 (61) | 3 (30) | 1 (25) | 2 (50) | 2 (33) |
| Pregabalin | 11 (26) | 1 (6) | 4 (40) | 3 (75) | 2 (50) | 1 (17) |
| Topical immunosuppressives | 0 (0) | 0 (0) | 0 (0) | 0 (0) | 0 (0) | 0 (0) |
| Topical corticosteroids | 27 (64) | 10 (56) | 8 (80) | 3 (75) | 3 (75) | 3 (50) |

**Abbreviations:** ADC, antibody-drug conjugate; CPI, checkpoint inhibitor; PI3K, phosphoinositide 3-kinase; TKI, tyrosine kinase inhibitor.

**Supplementary Figure 1. Clinical Photos and Corresponding Histology of Eosinophil-Related Cutaneous Adverse Event, Pre and Post-Treatment with Benralizumab**


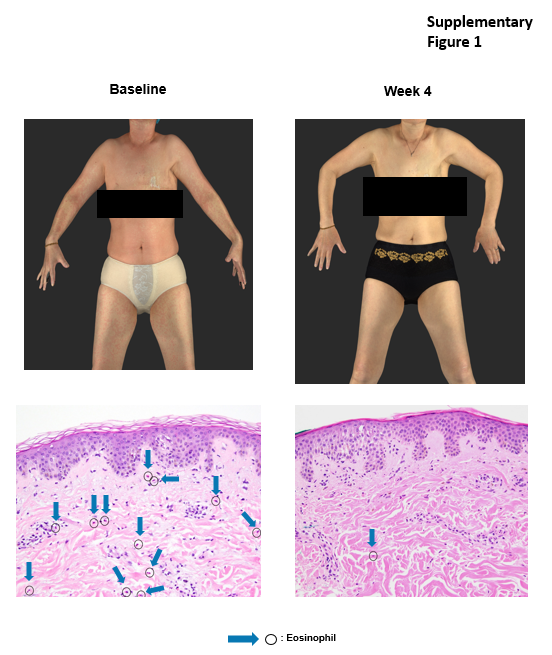


**Supplementary Figure 2. Relative Dose Intensity of Culprit Agent While on Study, Categorized by Culprit Agent**


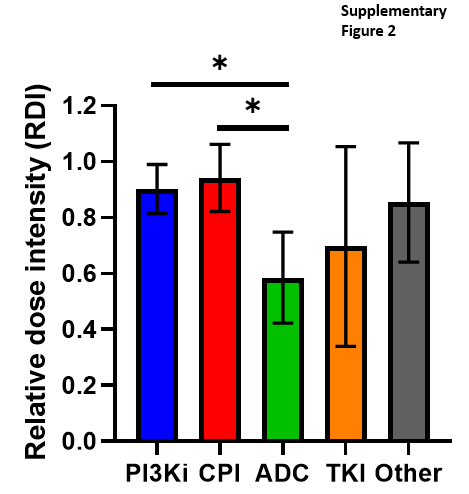


ADC indicates antibody-drug conjugate; CPI, checkpoint inhibitor; PI3K, phosphoinositide 3-kinase; TKI, tyrosine kinase inhibitor. *, p<0.05.
